# Comparison of the efficacy of curcumin and its nano formulation on dexamethasone-induced hepatic steatosis, dyslipidemia, and hyperglycemia in Wistar rats

**DOI:** 10.1016/j.heliyon.2024.e41043

**Published:** 2024-12-06

**Authors:** Amany M. Hamed, Dalia A. Elbahy, Ahmed RH. Ahmed, Shymaa A. Thabet, Rasha Abdeen Refaei, Islam Ragab, Safaa Mohammed Elmahdy, Ahmed S. Osman, Azza MA. Abouelella

**Affiliations:** a Chemistry Department, Faculty of Science, Sohag University, Sohag, Egypt; b Department of Clinical Pharmacology, Faculty of Medicine, Sohag University, Sohag, Egypt; c Department of Pathology, faculty of medicine, Sohag University, Sohag, Egypt; d Central Research Center, Faculty of Medicine, Sohag University, Sohag, Egypt; e Department of Physiology, Faculty of Medicine, Sohag University, Egypt; f Department of Chemistry, College of Science, Qassim University, Buraidah, 51452, Saudi Arabia; g Department of Anatomy, Faculty of Medicine, Sohag University, Sohag, Egypt; h Department of Biochemistry, Faculty of Veterinary Medicine, Sohag University, Sohag, Egypt

**Keywords:** Type 2 diabetes, Nano-curcumin, Liver steatosis, Insulin resistance, Antihyperglycemic, Antihyperlipidemic

## Abstract

**Background and objective:**

Insulin resistance is a primary feature of type 2 diabetes. This study compared the effects of curcumin and its nanoformulation on insulin resistance, fasting blood sugar, liver function, GLUT4, lipid profile, and oxidative stress in the liver and pancreas in a diabetic model.

**Methods:**

Thirty male Wistar rats were divided into five groups: a control group, a diabetic group, a diabetic group treated with metformin (40 mg/kg), a diabetic group treated with curcumin (100 mg/kg), and a diabetic group treated with curcumin NPs (100 mg/kg). Diabetes was induced by injecting dexamethasone daily for 14 days. Treatment with curcumin and curcumin NPs was administered by gavage for 14 days. Body weight and fasting blood sugar levels were measured on days 1, 14, and 28.

**Results:**

The metformin, curcumin, and curcumin NPs groups showed significantly greater body weight gain than the untreated diabetic group (P < 0.001). In diabetic rats treated with curcumin and curcumin NPs, insulin resistance decreased by approximately 40 %, while fasting blood sugar levels dropped by 35–40 % (P < 0.001). The levels of liver enzymes (AST, ALT), cholesterol, triglycerides, LDL, and the oxidative stress marker MDA in liver and pancreatic tissues were reduced by 30–50 %. Additionally, beneficial markers such as albumin, HDL, antioxidants (GSH, SOD), and GLUT4 levels were increased by 25–45 % (P < 0.001). Nano-curcumin consistently showed greater improvements than curcumin, especially in reducing oxidative stress and supporting liver and pancreatic health.

**Conclusion:**

This study demonstrates that curcumin NPs has a superior effect on reducing oxidative stress and improving metabolic parameters in diabetes compared to curcumin. by enhancing the bioavailability and stability of curcumin, the nanoformulation showed stronger therapeutic potential for managing high blood sugar, cholesterol issues, and liver health, positioning curcumin NPs as a promising alternative to conventional treatments for diabetes and its complications.

## 1 Introduction

Type 2 diabetes mellitus (T2DM) is the most prevalent type of disease, accounting for about 80 % of cases of diabetes diagnosis. Diabetes is one of the top five causes of death worldwide [1]. The global public health danger of type 2 diabetes poses a threat to the economies of all countries, especially those in developing nations. Driven by quick development, dietary changes, and progressively inactive lifestyles, the epidemic has expanded simultaneously with the global surge in obesity cases [2]. The majority of patients with type 2 diabetes and obesity have insulin resistance in their muscles, liver, and fat, which reduces these tissues' sensitivity to insulin [3].Given the rising prevalence of type 2 diabetes and its association with glucocorticoid use, understanding the underlying mechanisms and potential intervention is crucial.

The action of glucocorticoids in target tissues is influenced by the density of nuclear receptors and the intracellular metabolism facilitated by two isozymes of 11β-hydroxysteroid dehydrogenase (11β-HSD). These isozymes catalyze the reversible conversion between active glucocorticoids, cortisol, and corticosterone, and their inactive forms, cortisone, and 11-dehydrocorticosterone. 11β-HSD1 typically functions as an oxidase (dehydrogenase) in vitro, converting active cortisol (in humans) or corticosterone (in rodents) into their inactive forms, cortisone and 11-dehydrocorticosterone, respectively. However, in intact cells, especially under specific physiological conditions, 11β-HSD1 acts predominantly as a reductase, regenerating active glucocorticoids from inactive forms. This reversal to reductase activity is indeed central to enhancing glucocorticoid action at the cellular level, often influenced by factors like cofactor availability (NADPH) and cellular context. 11β-HSD1 is widely expressed in the gonads, adult brain, inflammatory cells, liver, adipose tissue, muscle, and pancreatic islets. In obesity, 11β-HSD1 is primarily elevated in adipose tissue, which causes metabolic problems [4].

Patients with subclinical Cushing's syndrome have been reported to have a higher frequency of hypertension, central obesity, impaired glucose tolerance, diabetes, and hyperlipoproteinemia [5]. Since the 1940s, when glucocorticoid therapy for autoimmune disease was first introduced, the widespread use of these drugs has resulted in the concurrent discovery of numerous harmful metabolic side effects, which has limited therapy. Unexpected hyperglycemia caused by the start of glucocorticoids frequently results in avoidable hospital admissions, extended hospital stays, higher infection risks, and worsened graft function in recipients of solid organ transplants. The management of diabetes induced by steroids is complicated by the large ranges in post-prandial hyperglycemia and the absence of well-defined treatment guidelines [6,7]. Insulin resistance and hepatic steatosis are strongly correlated. Numerous reports discuss the connection between glucocorticoids and hepatic steatosis [8]. In mice models of non-alcoholic fatty liver disease (NAFLD) and non-alcoholic steatohepatitis disease (NASHD), metformin reverses steatosis, inflammation, and abnormalities of aminotransferases [9]. Metformin has been shown in numerous clinical trials to be beneficial for patients with NAFLD and NASHD [10,11]. Nevertheless, a meta-analysis found that metformin did not help non-alcoholic steatohepatitis (NASH) patients' steatosis, lobular inflammation, hepatocellular ballooning, or fibrosis [12]. According to recent guidelines, metformin is not advised as a specific treatment for liver disease in adults with NASHD because it does not significantly affect liver histology [13].

Numerous supplements derived from medicinal plants have been investigated for their possible advantages in treating hepatic steatosis. Curcumin, a phenolic compound derived from the turmeric root (Curcuma longa), may lower the hepatic fat content (HFC) in NAFLD, according to systematic reviews of the literature [14,15]. Furthermore, meta-analyses have demonstrated that curcumin can enhance several liver-related parameters in people with NAFLD, such as circulating levels of triglyceride, total and low-density lipoprotein cholesterol, alanine transaminase, and HbA1c, as well as fasting plasma glucose, hyperinsulinemia, insulin resistance (as measured by the homeostatic model assessment [HOMA-IR]), body weight, body mass index (BMI), and waist circumference [[16], [17], [18], [19]].

Moreover, curcumin appears to possess anti-inflammatory characteristics [20,21]. Curcumin influences multiple aspects of Metabolic Syndrome including insulin sensitivity, blood pressure, inflammation, and abiogenesis suppression [22]. However, Curcumin's low absorption from the gastrointestinal tract, bioavailability, and water solubility limit its beneficial effects [23], some curcumin nanoparticles have a significantly higher bioavailability than the simple powder form [24]. Nanotechnology-based pharmaceutical formulations, particularly those incorporating curcumin NPs, have emerged as promising solutions to enhance the bioavailability of curcumin and amplify its anti-diabetic properties. Conventional curcumin is hindered by its low solubility and rapid metabolic degradation, which limits its therapeutic effectiveness. However, by utilizing nanocarrier systems, researchers have significantly improved curcumin's absorption and stability, facilitating more efficient delivery to target tissues [25]. Previous studies have demonstrated that curcumin NPs can effectively improve key metabolic parameters in diabetic animal models, including substantial reductions in blood glucose levels and enhanced insulin sensitivity. Moreover, nano-curcumin possesses strong antioxidant and anti-inflammatory capabilities, addressing oxidative stress and chronic inflammation commonly associated with type 2 diabetes mellitus, poetries not only contribute to improved glycemic control but also help mitigate the long-term complications of diabetes, such as cardiovascular diseases and metabolic syndrome [26]. Curcumin and curcumin NPs have the same chemical structure, but theoretically, curcumin NPs could be just as effective as curcumin at reducing the risk factors for cardiovascular disease. For example, some earlier in vitro and in vivo research suggested that curcumin NPs might have some therapeutic benefits over native curcumin [27,28].

Ashtary-Larky et al., reported that curcumin NPs supplementation was associated with an improved glycemic profile by decreasing fasting blood glucose, fasting insulin, and HOMA-IR. Moreover, curcumin NPs supplementation resulted in a rise of HDL. These researchers also found decreases in C-reactive protein, and interleukin-6, which show the favorable anti-inflammatory and hypotensive effects of curcumin NPs supplementation [29].

Turmeric's bioactive ingredient curcumin has attracted attention as a possible treatment for type 2 diabetic mellitus (T2DM), mainly because of its insulin-sensitizing, anti-inflammatory, and antioxidant qualities. Examining curcumin's efficacy and mode of action is crucial when comparing it with better-developed therapies like metformin, sulfonylureas, and more recent ones like glucagon-like peptide-1 (GLP-1) receptor agonists. Glycemic management in type 2 diabetes is based on factors like metformin, which lowers hepatic glucose production and improves insulin sensitivity. While GLP-1 receptor agonists enhance insulin production in response to meals and decrease stomach emptiness, sulfonylureas enhance the release of insulin from pancreatic beta-cells, promoting appetite and weight control. Curcumin has a different mechanism of action, but it affects oxidative stress reduction and inflammatory pathways (including the NF-kB pathway), both of which are linked to insulin resistance. It has also been discovered that curcumin directly alters insulin signaling pathways, but the exact mechanisms are yet not fully known [30,31].

Studies on curcumin show small, variable effects on fasting glucose and HbA1c levels, whereas metformin and sulfonylureas consistently show considerable decreases in these parameters. Curcumin usually reduces fasting glucose and HbA1c by a smaller amount in trials, and the outcomes vary less among groups and research types [32]. At larger dosages, gastrointestinal problems are the main side effect of curcumin, which is generally considered safe [33]. In contrast to sulfonylureas, which may result in hypoglycemia, and GLP-1 agonists, which may cause nausea and pancreatitis, curcumin has a comparatively low incidence of side effects, which makes it a desirable therapy adjunct [34].

Most research on curcumin in T2DM involves comparisons with placebos rather than established antidiabetic drugs like metformin or GLP-1 receptor agonists. This makes it difficult to assess curcumin's relative effectiveness and to understand if it could serve as a substitute or supplement to existing therapies. Direct comparisons with these medications are essential for positioning curcumin within the spectrum of T2DM treatments, yet few studies have tackled this. Addressing this gap could reveal curcumin's value in T2DM management—whether as a complementary therapy or a viable alternative in certain cases [[35], [36], [37], [38]].

These gaps illustrate the limitations of current research on curcumin for T2DM, underscoring the need for more rigorous, standardized, and comprehensive studies. Until such research is conducted, curcumin's role in T2DM management remains tentative. Addressing these gaps through well-designed trials and mechanistic studies would provide a clearer, evidence-based foundation for integrating curcumin into T2DM treatment, whether as a primary option or as a beneficial adjunct.

Therefore, the primary objective of this study is to evaluate the effects of curcumin and its nanoformulation on insulin resistance and metabolic disorders in Wistar rats with dexamethasone-induced hyperglycemia and dyslipidemia. The research will compare the therapeutic efficacy of curcumin, its nanoformulation, and metformin while exploring their mechanisms for improving metabolic health.

## 2 Materials and methods

### 2.1 Materials and drugs

Chitosan (Low molecular weight), Sodium tripolyphosphate, Curcumin, Metformin, and Dexamethasone pharmaceutical grade were purchased from (Glentham - UK).

### 2.2 Reagents and kits

Levels of glucose, total cholesterol, triglycerides, high-density lipoproteins (HDL), low-density lipoproteins (LDL), albumin, and aminotransferases (AST and ALT) were measured using commercially available diagnostic kits from Bio-diagnostic (Cairo, Egypt). Serum Insulin levels were estimated by using an Ultra-sensitive rat insulin ELISA kit from Gen X Bio Health Sciences Private Limited, New Delhi. glutathione (GSH), superoxide dismutase (SOD, and malondialdehyde (MDA) detection kits were obtained from Nanjing Jiancheng Bio-Technology Co. Ltd (Nanjing, China). GLUT4 was obtained from Abcam (USA). The kit manufacturer's manual's instructions served as the basis for the measurement technique. These kits have been rigorously validated for experimental use and typically demonstrate high sensitivity, often exceeding 90 %. This high sensitivity ensures reliable detection of metabolic markers, which is essential for accurate diagnosis and monitoring of conditions like diabetes and dyslipidemia.

### 2.3 Preparation of nanoformulation of curcumin

Curcumin-loaded chitosan NPs (CUR NPs) were prepared with a slight modification of previously reported ionic gelation method by Duse et al. [39]. CUR NPs were created at Sohag University in Egypt's Faculty of Science. Dimethylsulfoxide (DMSO) was used to dissolve curcumin while stirring continuously. Chitosan was dissolved in glacial acetic acid at room temperature, diluted with distilled water, and combined with the curcumin solution using a magnetic stirrer (Thermolyne, USA) running at 500 rpm. To create nanoparticles, the mixture was then dripped with tripolyphosphate (TPP) at a rate of one drop every 3 s using a burette and a 500-rpm magnetic stirrer. The mixture was then left over the magnetic stirrer for half an hour to produce a stable curcumin nanoparticle solution. After being separated by an ultracentrifuge (Hanil Micro 17 TR centrifuge - HE5) for 30 min at 4 °C and 17000 rpm, the nanoparticles were lyophilized (freeze-dried) and kept at 4 °C for use in subsequent studies. The stability of the curcumin nanoparticles was then monitored for five days, along with their color, turbidity, and sedimentation. Fourier transform infrared spectroscopy (FT-IR), drug loading capacity (DLC), entrapment efficiency (EE), average particle size and size distribution, and transmission electron microscope images were used to determine the characteristics of the CUR NPs.

### 2.4 Evaluation and characterization of curcumin nano nanoparticles

#### 2.4.1 Particle size, distribution nanoparticles, and morphology examination

Using the JEOL JEM 100 CXII (100 KV), the curcumin nanoparticle's surface morphology, microscopic structure characterization, and particle size and distribution were all examined.

#### 2.4.2 Fourier transform infrared spectroscopy (FTIR)

A popular method for analyzing the structure of molecules, identifying chemical bonds between them, and defining their structure is FTIR spectroscopy. Across the FTIR spectra absorption band, certain functional groups present in the molecular chemical structure are resolved. Interactions between various substances and medications were noted in the NPs. Using (ATR-FTIR, Alpha Bruker Platinum, 1-211-6353), the FTIR spectra of the chitosan NP sample were determined.

#### 2.4.3 Determination of EE and DLC of curcumin nanoparticles

After curcumin loading, nanoparticles were separated from the suspension by ultracentrifugation (Hanil Micro 17 TR centrifuge - HE5) at 17000 rpm and 4 °C for 30 min. The amount of free curcumin in the supernatant was measured by UV-spectrophotometer at wavelength 422.5 nm. The Encapsulation efficiency (EE) and drug loading capacity (DLC) of nanoparticles were calculated using the following equations:

Encapsulationefficiency(%)=[(T−F)/T]×100

Drugloadingcapacity(%)=[(T–F)/W]×100

Where **F** is the free amount of curcumin (Non encapsulated curcumin) in the supernatant (mg), **T** is the total amount of curcumin added into the chitosan solution (mg), and **W** is the weight of nanoparticles (mg).

### 2.5 Experimental animals

Healthy Wistar male rats weighing around 155–184 gm were used in the present study. The Sohag Institutional Animal Care and Use Committee (Sohag- IACUC) of the Faculty of Medicine at Sohag University provided ethical approval for this research, which was carried out by protocol no. Sohag-5.November 5, 2023.03. The experimental animals' discomfort and suffering were kept to a minimum during every step of the process. The rats were acclimated for two weeks to the following conditions: 25 °C ± 2 °C, 65 % ± 10 % humidity, 11–13 air ventilation cycles per hour, and 12 h of light per day. Standard pellets were supplied to the rats, and they also received unlimited access to water.

### 2.6 Experimental methods

A total of 30 rats were divided into 5 groups with 6 rats in each group. The rats were divided according to weight into (160–167, 180–184, 170–180, 150–162, and 158–169 gm) for groups 1, 2, 3, 4, and 5 respectively. Bodyweight was checked for all groups on day 1, day 14, and day 28. Fasting blood glucose was checked for all groups on days 1, 10, 14, and 28. Insulin resistance was induced by intraperitoneal injection of dexamethasone (1 mg/kg) for 14 days in all treated groups. Animals were fasted overnight (14 h) before dexamethasone treatment as described by Mahendran and Devi [40].

**Group 1** served as normal control and rats were given the vehicle (DMSO: Tween 80: Water) in a volume ratio of 1:1:8 [41]**.**

**Group 2** served as dexamethasone (DEXA) control received dexamethasone alone.

**Group 3** served as positive control and received an oral reference drug (Metformin, MET) (40 mg/kg) for 14 days after dexamethasone injection.

**Groups 4 and 5** were treated orally with (Curcumin, CUR) and curcumin NPs (CUR NPs) respectively [42] at a dose of 100 mg/kg for 14 days after dexamethasone injection.

Rats were anesthetized and then sacrificed by cervical dislocation. The liver and pancreas were dissected out. Selected organs were stored in 10 % formalin and sent for histopathological analysis.

### 2.7 Blood sampling for biochemical analysis

After obtaining a clear serum by centrifuging blood samples for 10 min at 3000 rpm, the samples were kept at −20 °C for biochemical analysis. Samples of tissue from the pancreas and liver have been sliced and prepared for histology and biochemical analysis.

### 2.8 Body weight, blood glucose, lipid, and insulin levels

All rats had their fasting blood glucose (FBG) levels tested with an Accu-Chek meter (Roche Diagnostics GmbH, Mannheim, Germany) from the tail vein. For each time point, three measurements were made. We recorded measurements of their body weight on day 1, day 14, and day 28. Following rats sacrificed by cervical dislocation, blood was collected from the heart. After the blood was centrifuged, a lipid profile was assessed in the serum by measuring the levels of triglycerides, high-density lipoproteins (HDL), low-density lipoproteins (LDL), and total cholesterol. An assay kit for rat insulin enzyme-linked immunosorbent was used to measure the levels of fasting serum insulin (FSI). The formula for the insulin resistance index (IRI) is IRI = FBG × FSI/22.5 [43]**.**

### 2.9 Determination of liver function tests

A Hitachi Analyzer Model 911 (Hitachi) was used to measure the serum activities of albumin, aspartate (aminotransferase, AST), and (alanine aminotransferase, ALT) in rats.

### 2.10 Determination of oxidative stress

The pancreas and liver of every rat were taken out immediately and weighed. Each rat's portion of the pancreas and liver were homogenized in a glass homogenizer using cold phosphate-buffered saline (1:4) (pH 7, 0.01 mol/L) containing a protease/phosphatase inhibitor cocktail (Cat. No. PPC1010, **Sigma**-Aldrich, USA). The resulting homogenate was filtered, centrifuged at 5000×*g* for 5 min, and then used to assess oxidative stress markers such as GSH, SOD, and MDA. Three repeats of the trials were conducted [44].

### 2.11 Detection of GLUT4

Muscles were separated from connective tissue, liquid nitrogen was frozen quickly, and then kept at −70 °C for additional analysis, and it was weighed. A glass homogenizer was used to homogenize the muscle component of each rat, using cold phosphate-buffered saline (1:4) (pH 7, 0.01 mol/L). After filtering and centrifuging at 5000×*g* for 5 min, the homogenate was utilized to measure GLUT4. The trials were carried out three times [44].

### 2.12 Histopathological studies

For preparation of histological sections of different investigated groups; tissue samples of the liver and pancreas were fixed in 10 % neutral buffered formalin for 24–36 h at room temperature. The tissue samples underwent the following steps: dehydration at room temperature by upgraded ethyl alcohol, clearance at room temperature by xylene, and embedding in paraffin at 70 °C. After the construction of paraffin blocks, tissue sections of 5 μm thicknesses were de-paraffinized in xylene, re-hydrated by downgraded ethyl alcohol, and washed in running tap water. For hematoxylin and eosin staining, the sections were incubated in hematoxylin for 7 min at room temperature, washed in running water, and incubated in eosin for 2 min. The sections were washed in running tap water, dehydrated by upgraded ethyl alcohol, cleared in xylene, and mounted using Dibutylphthalate Polystyrene Xylene (DPX).

### 2.13 Immunohistochemical studies

Four micrometer sections of formalin-fixed and paraffin-embedded tissue blocks of the liver were de-paraffinized by xylene; followed by rehydration in downgraded alcohol and washing in running water. For blocking of endogenous peroxidase activity, tissue sections were incubated in 3 % H2O2 for 10 min at room temperature. Antigen retrieval was performed by incubation of tissue sections in 0.01 mmol/L Citrate buffer solution (pH 6) at 92 °C for 20 min. After washing by PBS buffer, tissue sections were incubated with either anti- Tumor necrosis factor (TNF) mouse monoclonal antibody (Clone 52B83, Catalog #NB600-1422, Novus Biologicals) or anti- Proliferating Cell Nuclear Antigen (PCNA) mouse monoclonal antibody (Clone PC 10, Catalog # NB 500, Novus Biologicals) for 1 h at room temperature. Tissue sections were washed twice with PBS before incubation with goat anti-mouse secondary antibody, incubated in streptavidin-biotin for 10 min, and separated by washing in BPS for 5 min after each step. The reaction products were visualized by immersing the sections in diaminobenzidine (DAB) for 15 min at room temperature (ScyTek, P.O. Box 3286- Logan, Utah 84323, USA). Nuclear counterstaining was done by immersion in Harris’ Hematoxylin for 2 min followed by rapid washing in tap water to remove extra dye. Sections were dehydrated by upgraded alcohol, cleared in xylene, and mounted using DPX. TNF and PCNA expression were evaluated based on a percentage of positive cells regarding the intensity of immune staining [45]. Evaluation of histological and immune-stained sections was performed using a binocular Olympus microscope CX40 RF200 (Olympus Optical Co., LTD).

### 2.14 Statistical analysis

Results were analyzed by one-way ANOVA followed by Tukey multiple comparison tests using SPSS software (version 27). The values in each group are characterized by a normal distribution and identical variance, the data was presented in Mean ± SD. For the non-parametric data (Body Weight and Fasting Blood Glucose), the equality of groups’ means was additionally checked by the Kruskal-Wallis one-way ANOVA by ranks and multiple comparison tests, the data was presented in Mean ± SE. Statistical significance was assumed if P < 0.05.

## 3 Results

### 3.1 Curcumin-loaded chitosan nanoparticle characterization

#### 3.1.1 Stability of curcumin nanoparticles

Color, turbidity, and sedimentation of the curcumin nanoparticles were observed for five days following formulation to assess their stability. We discovered that the turbidity remained constant, there was no color shift, and there were no deposits of curcumin at the vial's bottom.

#### 3.1.2 Transmission electron microscopy (TEM)

• Particle Size Distribution

**Fig. 1:**
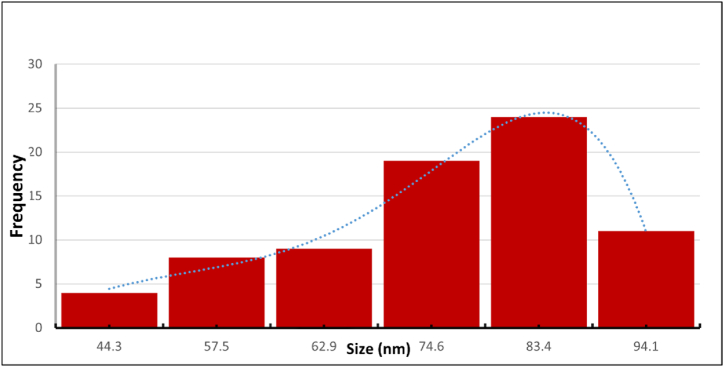
Histogram of Particle size distribution curve of CUR NPs from TEM**.**

The curcumin-loaded chitosan nanoparticles' particle size histogram (Fig. 1) reveals that the particles' median size is 68.75 nm, with a range of 44.3–94.1 nm. The histogram's size distribution reveals that about 80 % of the particles fall between 74.6 and 83.4 nm.

• TEM morphology

**Fig. 2:**
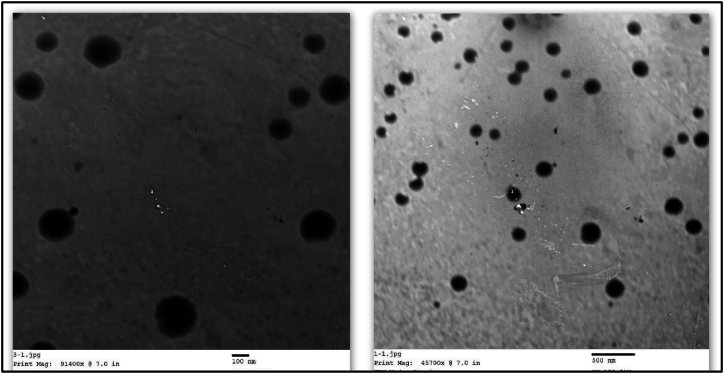
TEM images of CUR NPs (scale bar 100 and 500 nm) showing microspheres.

To determine the morphology and shape of the nanoparticles, TEM measurements were performed. The TEM Nano graphs (Fig. 2) magnified to 100 and 500 nm scales showed that the chitosan nanoparticles loaded with curcumin have a homogeneous distribution, spherical shape, and homogenous structure.

#### 3.1.3 Fourier-transform infrared (FT-IR) analysis

**Fig. 3:**
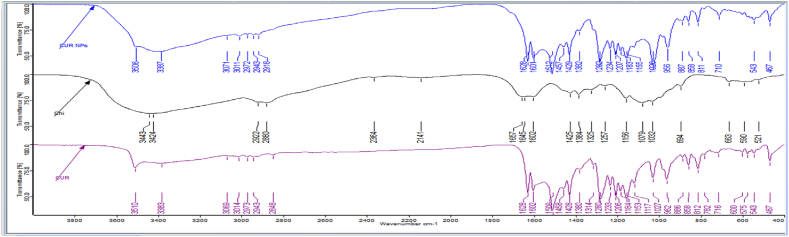
FTIR analysis of chitosan (Chi), curcumin, and curcumin nanoparticles (CUR NPs).

Fig. 3 shows that the curcumin spectrum (purple color) had two characterization peaks (1117 cm^−1^ of (C-O-C) and 1509 cm^−1^ of (OH)), whereas the chitosan spectrum (black color) showed three characterization peaks (1079 cm^−1^ of (C-O-C), 1424 cm^−1^, and 1383 cm^−1^ of (NH2)). When curcumin-loaded chitosan-TPP nanoparticles (blue color) were compared to curcumin, a distinct spectrum was seen, with new, strong peaks emerging at 3387 cm^−1^ and 1026 cm^−1^. Additionally, the peak vibration of 1509 cm^−1^ shifted to 1511 cm^−1^. It is possible that in the nanoparticles, the hydroxide groups of curcumin and the ammonium groups of chitosan were connected. The previous study on curcumin loading into chitosan nanoparticles revealed similar findings [46,47].

#### 3.1.4 EE and DLC of curcumin loaded Chitosan-TPP nanoparticles

Following the chitosan-TPP nanoparticles loaded with curcumin being prepared, the nano-formulation was collected and centrifuged. The remaining curcumin in the solution's supernatant was then quantified using a spectrophotometer (UviLine 9400) with a wavelength of 422.5 nm. For curcumin, the results showed that the encapsulation efficiency (EE%) and drug loading capacity (DLC%) were 97.67 % and 52.87 %, respectively.

### 3.2 Effect of curcumin/curcumin NPs on body weight

Table 1 displays the five groups' body weights. In comparison to day 28, the dexamethasone group showed a decrease in weight. When comparing the curcumin, and curcumin NPs treated groups to the dexamethasone control group, a significantly higher increase in body weight was noted (P < 0.001). The similarity in weight gain between curcumin NPs and metformin groups (P = 0.865) suggests that nano-curcumin may offer comparable metabolic benefits to metformin in countering diabetes-induced catabolic effects. Including an effect size, such as Cohen's d, for weight gain differences between treatment groups and the dexamethasone, control could quantify this outcome more precisely.

**Table 1:** Effect of Curcumin and Curcumin NPs on body weight in dexamethasone-induced Rats (n = 6 per group).

Groups	Body weight (gram)		
	Day1	Day14	Day28
Normal Control	164.8 ± 1.0	195.6 ± 3.0	230.6 ± 3.9
Dexamethasone Control	181.5 ± 0.7a	132.6 ± 3.0a	133.5 ± 3.2a
Metformin	177.0 ± 1.5	129.8 ± 1.9	200.6 ± 2.1
Curcumin	157.3 ± 1.7	131.3 ± 2.0	204.0 ± 1.0bc
Curcumin NPs	162.8 ± 2.1	129.8 ± 1.8	204.0 ± 0.8bc

Results were analyzed by Kruskal-Wallis one-way ANOVA, Values are mean ± SE.

P < 0.001 vs. Normal control.

P < 0.001 vs. Dexa Control group.

P < 0.001 vs metformin group.

### 3.3 Effect of curcumin/curcumin NPs on fasting blood glucose

Table 2 shows the blood glucose levels of the five groups after a fast. Day 28 showed an elevation in fasting blood glucose levels in the dexamethasone group relative to the control group. As compared to the dexamethasone control group, there was a highly significant drop in the fasting blood glucose levels in the groups treated with metformin, curcumin, and curcumin NPS (P < 0.001). The groups treated with curcumin and curcumin NPS had fasting blood glucose levels that were like those of the metformin group (P = 0.250/0.949 respectively). This effect highlights the potential of curcumin, particularly in its nanoform, to act as a glucose-lowering agent. Reporting the percent reduction in fasting blood glucose and calculating effect sizes like Hedges' g could provide a clearer picture of nano-curcumin's relative efficacy.

**Table 2:** Effect of Curcumin and Curcumin NPs on fasting blood glucose levels in dexamethasone-induced Rats (n = 6 per group) Fasting blood glucose level (mg/dl).

Groups	Day1	Day10	Day14	Day28
Normal Control	102.6 ± 2.0	101.8 ± 2.0	100.8 ± 1.3	92.2 ± 4.8
Dexamethasone Control	106.8 ± 3.4	128.0 ± 0.8^a^	145.1 ± 1.1^a^	140.1 ± 3.1^a^
Metformin	103.1 ± 2.2	128.6 ± 0.8	144.5 ± 1.2	101.3 ± 6.9
Curcumin	101.5 ± 1.5	128.0 ± 0.5	143.5 ± 1.7	102.0 ± 1.2^b^
Curcumin NPs	104.3 ± 2.4	128.3 ± 0.8	146.3 ± 0.5	103.0 ± 3.6^b^

Results were analyzed by Kruskal-Wallis one-way ANOVA, Values are mean ± SE.

a P < 0.001 vs. Normal control.

b P < 0.001 vs. Dexa Control group, c P < 0.001 vs metformin group.

### 3.4 Effect of curcumin/curcumin NPs on lipids

Table 3 summarizes the lipid profiles of the five groups. The dexamethasone group exhibited elevated levels of cholesterol, triglycerides, and LDL, and decreased HDL in contrast to the normal group. Compared to the dexamethasone control group, Curcumin and curcumin NPs markedly improved lipid profiles, reducing cholesterol, triglycerides, and LDL while increasing HDL levels (P < 0.001). Interestingly, the curcumin NPs group showed greater lipid-lowering effects than metformin (P < 0.001), indicating superior efficacy in managing dyslipidemia. Presenting confidence intervals for the changes in lipid levels and calculating effect sizes would reinforce the clinical importance of these improvements, especially since lipid control is crucial in diabetes management to prevent cardiovascular complications.

**Table 3:** Effect of Curcumin and Curcumin NPs on lipids in dexamethasone-induced Rats (n = 6 per group).

Groups	CH (mg/dl)	TGS (mg/dl)	HDL (mg/dl)	LDL (mg/dl)
Normal Control	93.5 ± 1.03	55.9 ± 0.7	27.8 ± 0.6	54.5 ± 0.9
Dexamethasone Control	206.3 ± 1.01a	173.3 ± 2.6a	5.3 ± 0.3a	166.2 ± 1.1a
Metformin	121.6 ± 1.15	81.2 ± 0.9	22.5 ± 0.5	82.7 ± 0.6
Curcumin	139.6 ± 0.2bc	90.8 ± 0.4bc	21.9 ± 0.9b	99.4 ± 0.9bc
Curcumin NPs	101.3 ± 2.4bc	93.2 ± 0.3bc	25.7 ± 0.08b cc	57.0 ± 2.4bc

Values are mean ± SD, CH = Cholesterol, TGS = Triglycerides, HDL = High-density lipoproteins, and LDL = Low-density lipoproteins.

P < 0.001 vs. Normal control.

P < 0.001 vs. Dexa Control group.

P < 0.001 vs metformin group.

### 3.5 Effect of curcumin/curcumin NPs on liver function parameters

**Fig. 4:**
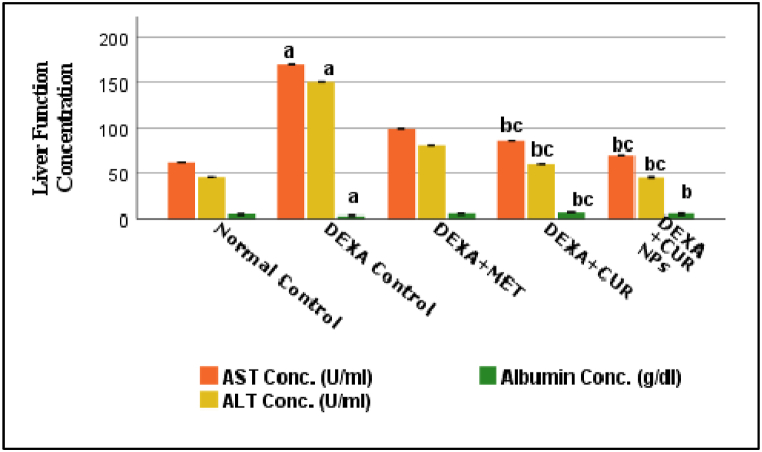
Effect of Dexa, Metformin, CUR, and CUR NPs on liver function parameters (AST, ALT, Albumin), (Results were analyzed by One-way ANOVA and Tukey's post hoc tests. Results are shown in mean ± SD (n = 6). ^a, b, c^ p < 0.001 compared to Normal control, Dexa Control, and Metformin groups, respectively.

The impact of the five groups' liver function parameters is shown in Fig. 4. Compared to the normal group, the dexamethasone group had higher levels of AST, and ALT, and smaller levels of albumin. The AST, and ALT levels were significantly lower in the metformin, curcumin, and curcumin NPs treatment groups than in the dexamethasone control group while the albumin level was significantly higher in the metformin, curcumin, and curcumin NPs treatment groups than in the dexamethasone control group (P < 0.001). The AST, ALT, and Albumin levels in the curcumin/curcumin NPs treated groups were comparable to those in the metformin group (P < 0.001/P < 0.001, P = P < 0.001/P < 0.001, and P < 0.001/P = 0.932, respectively). This indicates that underscores nano-curcumin's role in supporting liver health. Adding effect sizes for AST, ALT, and albumin changes relative to the dexamethasone group could illustrate the magnitude of liver protection provided by curcumin NPs.

### 3.6 Effect of curcumin/curcumin NPs on liver and pancreas tissue homogenate antioxidant parameters

In Table 4, which illustrates oxidative stress, the administration of dexamethasone resulted in a significant (P < 0.001) reduction in the levels of antioxidant enzyme SOD and GSH marker as well as an increase in the lipid peroxidation marker MDA in the liver and pancreatic homogenate of dexamethasone control animals. When compared to the dexamethasone control group rats, treatment with metformin, curcumin, and curcumin NPs restored GSH level and SOD enzyme activity with a decline in MDA level with nano-curcumin showing superior effects. This indicates that nano-curcumin effectively counters oxidative stress in liver and pancreatic tissues, which is a critical component of diabetes complications. Including a comparison of mean percent changes and confidence intervals for these markers would emphasize nano-curcumin's protective effects on tissue health.

**Table 4:** Effect of Curcumin and Curcumin NPs on liver and pancreas tissue antioxidant parameters in dexamethasone-induced Rats (n = 6 per group).

**Groups**	MDA (Nmol/g tissue)		GSH (Pg/g tissue)		SOD (U/g tissue)	
	liver	Pancreas	Liver	Pancreas	liver	Pancreas
Normal Control	8.1 ± 0.07	6.7 ± 0.05	48.1 ± 0.08	38.2 ± 0.05	41.2 ± 0.09	31.6 ± 0.00
Dexamethasone Control	36.1 ± 0.05a	25.8 ± 0.05a	17.6 ± 0.1a	14.4 ± 0.04a	15.1 ± 0.07a	11.5 ± 0.06a
Metformin	28.7 ± 0.08	20.7 ± 0.05	20.3 ± 0.08	17.1 ± 0.05	17.5 ± 0.1	15.5 ± 0.08
Curcumin	17.4 ± 0.1bc	15.2 ± 0.05bc	31.7 ± 0.1bc	26.8 ± 0.06bc	30.2 ± 0.05bc	23.4 ± 0.04bc
Curcumin NPs	10.5 ± 0.05bc	10.9 ± 0.1bc	40.1 ± 0.1bc	32.7 ± 0.00bc	34.7 ± 0.05bc	28.06 ± 0.08bc

Values are mean ± SD, MDA = Malondialdehyde, GSH = Glutathione, SOD = superoxide dismutase.

P < 0.001 vs. Normal control.

P < 0.001 vs. Dexa Control group.

P < 0.001 vs metformin group.

### 3.7 Effect of curcumin/curcumin NPs on glycemic parameters and GLUT4 muscle homogenate

**Fig. 5:**
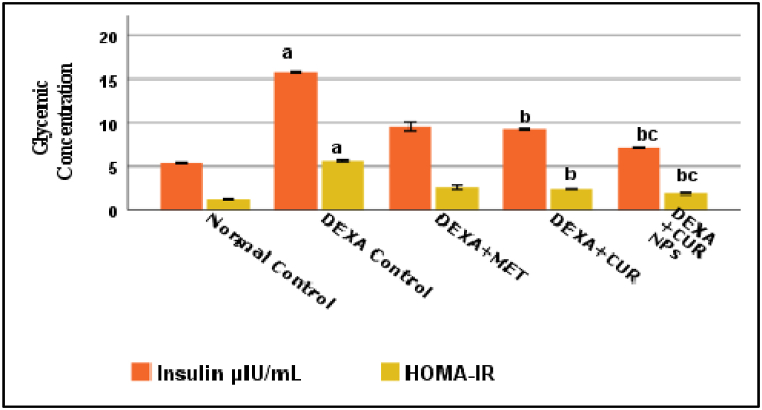
Effect of Dexa, Metformin, CUR, and CUR NPs on Glycemic concentration (Insulin and HOMA IR), (Results were analyzed by One-way ANOVA and Tukey's post hoc tests. Results are shown in mean ± SD (n = 6). ^a, b, c^ p < 0.001 compared to Normal control, Dexa Control, and Metformin groups, respectively.

**Fig. 6:**
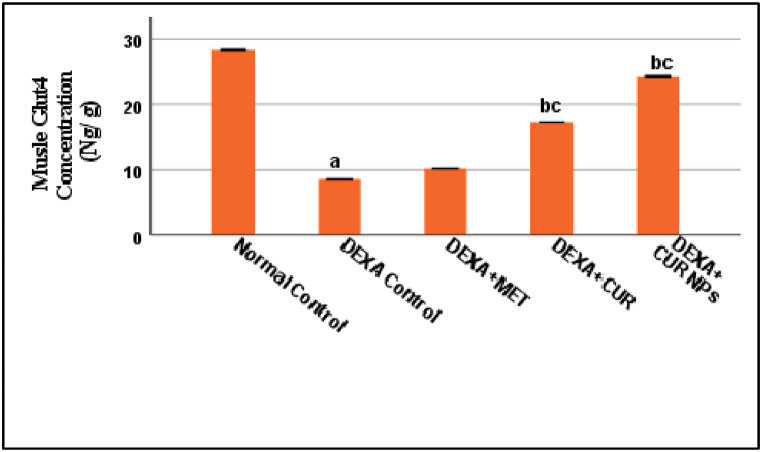
Effect of Dexa, Metformin, CUR, and CUR NPs on Muscles Glut4 concentration, (Results were analyzed by One-way ANOVA and Tukey's post hoc tests. Results are shown in mean ± SD (n = 6). ^a, b, c^ p < 0.001 compared to Normal control, Dexa Control, and Metformin groups, respectively.

The Glycemic Parameters and GLUT4 of the five groups are summarized in Fig. 5, Fig. 6. Dexamethasone significantly increased blood insulin and HOMAIR and decreased insulin-stimulated skeletal muscle glucose transport (p < 0.001). When compared to the dexamethasone group, these activities were significantly reversed by metformin, curcumin, and curcumin NPs. Curcumin NPs appeared to be more effective than curcumin and metformin in improving glycemic control (insulin, HOMAIR, and GLUT4). This suggests that nano-curcumin enhances insulin sensitivity and glucose transport, crucial for diabetes management. Adding effect sizes for HOMAIR and GLUT4 expression changes could quantify curcumin NPs impact on glycemic parameters more precisely.

### 3.8 Histopathological evaluation of liver tissue

**Fig. 7:**
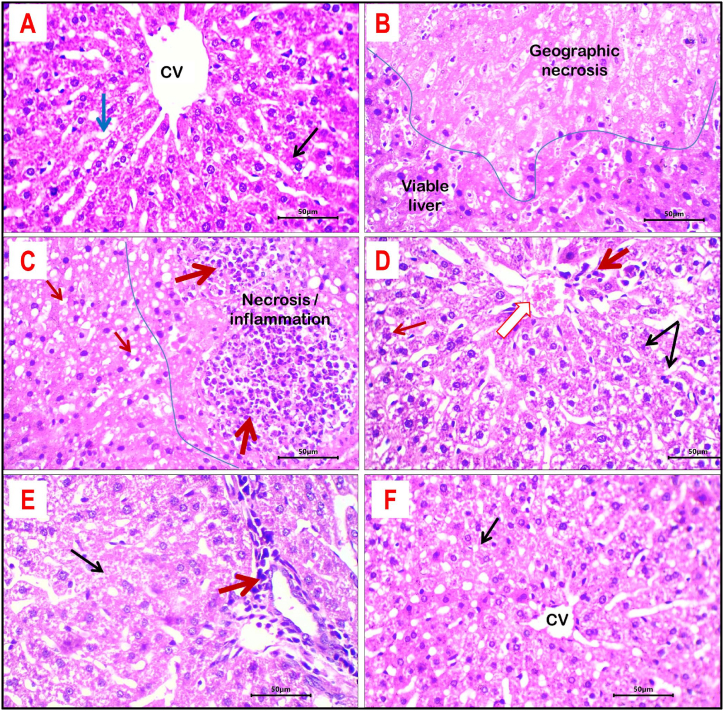
Histological sections of liver tissue from different study groups: **A-** Control group showed normal hepatic lobulation with preserved central vein (CV) and uniform hepatocyte cording (black arrow), separated by patent sinusoids (blue arrow). **B and C-** Liver tissue of dexamethasone-treated rats showed large areas of geographic necrosis (B) with dense inflammation (C, thick red arrows). Hepatocytes showed frequent micro-vesicular steatosis (thin red arrows). **D-** Rats treated with metformin showed mild degenerative changes (granular cytoplasm and cloudy swelling; black arrows), residual inflammation (thick red arrow), congested central vein (white arrow), and micro-vesicular steatosis (red arrow). **E-** Rats treated with curcumin showed residual portal inflammation (thick red arrow) and cloudy swelling of hepatocytes (black arrow). **F-** Treatment with nano-curcumin induced remarkable improvement of hepatic morphology with only residual focal mild cloudy swelling of hepatocytes (black arrow). H&E stained sections; magnification is ×400 for all.

For the control group, the liver showed preserved hepatic architecture with identified lobulation, central venules, and portal areas. Hepatocytes are arranged in 2 cell-thick plates with patent hepatic sinusoids. The liver cells have uniform size and shape with central nuclei (Fig. 7A). There is no evidence of hepatic degenerative changes, steatosis, necrosis, or inflammation.

Administration of dexamethasone-induced widespread damage of liver tissue (Fig. 7B and C). There are multiple foci of large geographic necrosis separated by zones of inflammation and degenerated liver tissue. Viable hepatocytes showed cloudy swelling, granular and glassy cytoplasm, and micro-vesicular and macro-vesicular steatosis. The central venules and hepatic sinusoids showed focal congestion. Hepatic lobules and portal areas are sears for patchy moderate inflammatory reactions mainly neutrophils and lymphocytes.

Treatment of rats with metformin-induced improvement of hepatic tissue damage changes compared to dexamethasone-treated rats. The necrotic effect of dexamethasone is very minimal (Fig. 7D). However, there is residual moderate venous and sinusoidal congestion and hepatocytes showed patchy degenerative changes namely micro-vesicular steatosis and cloudy swelling. Portal areas and hepatic lobules showed mild to moderate inflammation rich in lymphocytes.

Treatments of rats with either curcumin or nano-curcumin induced prominent improvement of the damaging effect of dexamethasone on liver tissue. Liver tissue of both curcumin and nano-curcumin-treated rats showed retained normal lobular architecture with normally appearing hepatic cording and almost absent steatosis. Residual histological changes include mild focal cloudy swelling and mild portal inflammation in liver tissue of curcumin-treated rate and mild cloudy swelling in liver tissue of nano-curcumin-treated rats. The main histopathological changes of liver tissue in different groups are summarized in Table 5.

**Table 5:** Main histopathological findings of liver tissues in different study groups.

Parameter	Study group				
	Control	Dexamethasone	Metformin	Curcumin	Curcumin NPs
Necrosis	–	++++	+	–	–
Central veins and sinusoid congestion	–	+++	+	–	–
Steatosis	–	+++	+	–	–
Cloudy swelling	–	+++	+	+	+
Portal/lobular inflammation	–	++++	+	+	–

Absent (−), minimal (+), mild (++), moderate (+++), severe (++++).

### 3.9 Histopathological evaluation of pancreatic tissue

**Fig. 8:**
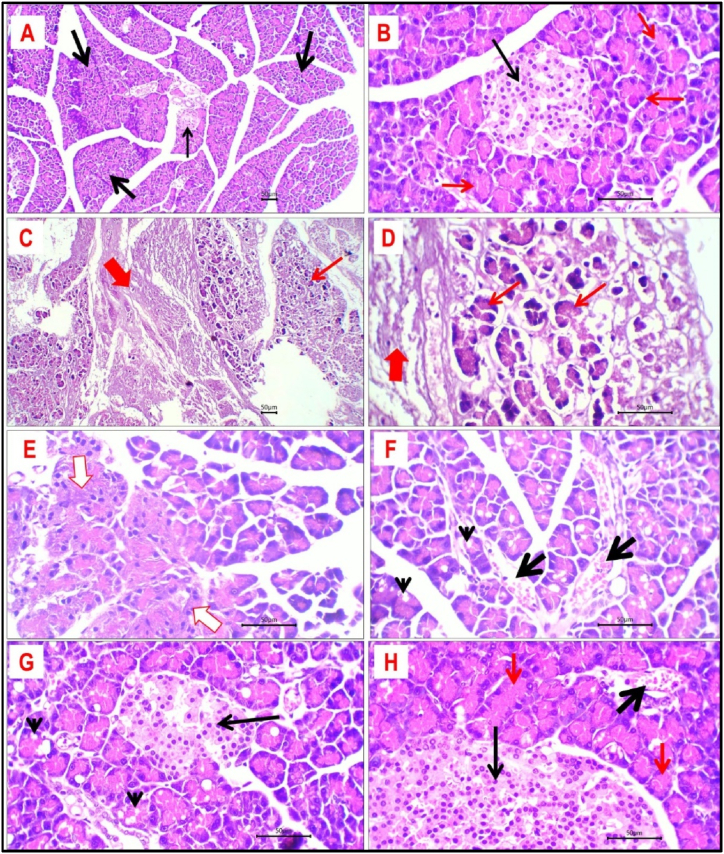
Histological sections of pancreatic tissue from different study groups: **A and B-** Control group: pancreatic tissue with preserved lobules (thick black arrows) and identified Islets' cells (thin black arrow). Pancreatic acini have uniform size and shape with uniform cuboidal cell lining (red arrows). **C and D-** Dexamethasone treated rats: Pancreatic tissue showed extensive necrosis (thick red arrows) with residual ghosts of pancreatic acini (thin red arrows). **E-** Metformin-treated rats: pancreatic tissue showed focal degeneration of pancreatic acini (white arrow). **F and G-** Curcumin-treated rats: there is residual cytoplasmic vacuolation of pancreatic acini (arrowhead) and multiple congested vessels (thick black arrows). **H-** Nano-curcumin-treated rats: Pancreas showed normal architecture of both exocrine (red arrows) and Islets' cells (thin black arrow) with few congested vessels (thick black arrow). H&E stained sections; magnification is ×100 for A and C and ×400 for others.

Histological sections of pancreatic tissue obtained from the control group showed normal lobulation of the pancreas with preserved exocrine and endocrine components (Fig. 8 A and 8 B). Pancreatic acini look uniform in size and shape. They are lined by a single layer of cuboidal cells with eosinophilic cytoplasm and have uniform nuclei. Multiple small aggregates of Islets’ cells were identified. They have a round to oval uniform shape with pale granular cytoplasm and uniform central nuclei. The stroma between Islets' cells contains a thin capillary network.

Pancreatic tissue of dexamethasone-treated rats showed widespread necrosis and degeneration. Necrotic areas appeared as zones of structureless eosinophilic tissue alternating with ghosts of acini and ghosts of Islets' cells (Fig. 8C and 8 D). No cellular or nuclear details could be detected.

Treatment with metformin showed remarkable improvement of pancreatic tissue with markedly reduced necrosis. Pancreatic tissue showed focal mild degeneration of exocrine pancreatic acini with almost viable Islet cells. Residual congested vessels and patchy mild stromal inflammatory reactions were observed (Fig. 8 E).

Treatment of rats with both curcumin and nano-curcumin induced prominent improvement of pancreatic tissue with absent necrosis. Pancreatic tissue retained normal architecture of both exocrine and endocrine components. For curcumin-treated rats (Fig. 8 F and 8 G); pancreatic tissue showed focal mild degeneration of exocrine pancreatic acini in terms of cytoplasmic cloudy swelling and cytoplasmic vacuolation. Islets' cells look viable with no recorded necrosis. Residual multiple congested vessels were identified. For nano-curcumin-treated rats (Fig. 8H); only a few congested vessels were seen with no evidence of residual necrosis, degeneration, or inflammatory reaction. The main histopathological changes of pancreatic tissue in different groups are summarized in Table 6.

**Table 6:** Histopathological findings of pancreatic tissues in different groups.

Parameter	Study group				
	Control	Dexamethasone	Metformin	Curcumin	Curcumin NPs
Necrosis/degeneration of the exocrine pancreas	–	++++	+	–	–
Necrosis/degeneration of the endocrine pancreas	–	++++	–	–	–
Inflammation	–	++	+	+	–

Absent (−), minimal (+), mild (++), moderate (+++), severe (++++).

### 3.10 Expression of TNF

**Fig. 9:**
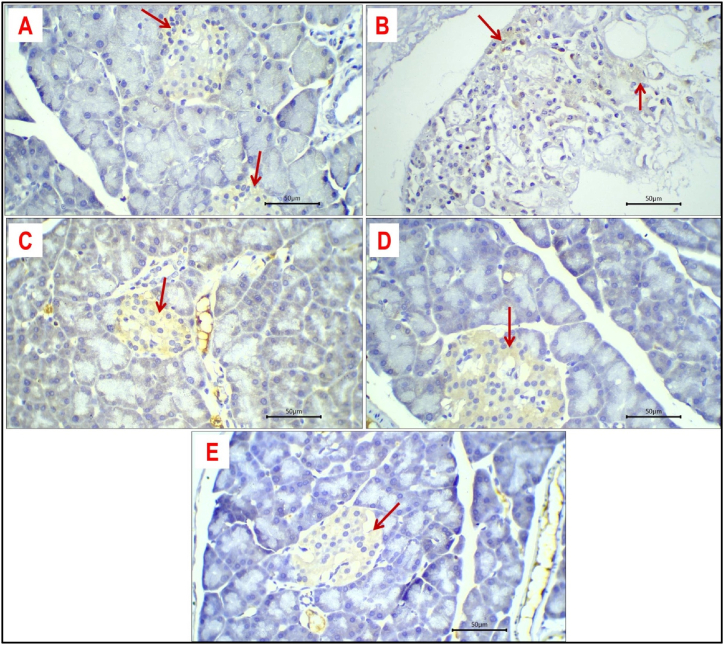
Expression of TNF in pancreatic tissue of different study groups: Faint to moderate cytoplasmic expression of TNF (red arrows) in pancreatic tissue of control group **(A),** dexamethasone-treated group **(B),** metformin-treated group **(C),** curcumin-treated group **(D)** and nano curcumin-treated group **(E).** Immune-stained sections; magnification is ×400 for all.

Expression of TNF was detected as brown, cytoplasmic staining. In general, expression of TNF is faint or weak to moderate in most positive cells of different investigated groups (Fig. 9). In addition, the expression was higher in islet cells compared to the exocrine pancreas. Expression of TNF was faint in the pancreatic tissue of the control group (Fig. 9 A) with sporadic positive cytoplasmic expression of TNF expression by scattered cells in other study groups. The average proportion of TNF-positive cells in the control group (Fig. 9 A), and dexamethasone-treated group (Fig. 9 B) is 1 % and 12 %; respectively and it was 8 %, 6 %, and 1 % in the metformin-treated group (Fig. 9C), curcumin-treated group (Fig. 9 D) and nano-curcumin treated group (Fig. 9 E); respectively.

### 3.11 Expression of PCNA

**Fig. 10:**
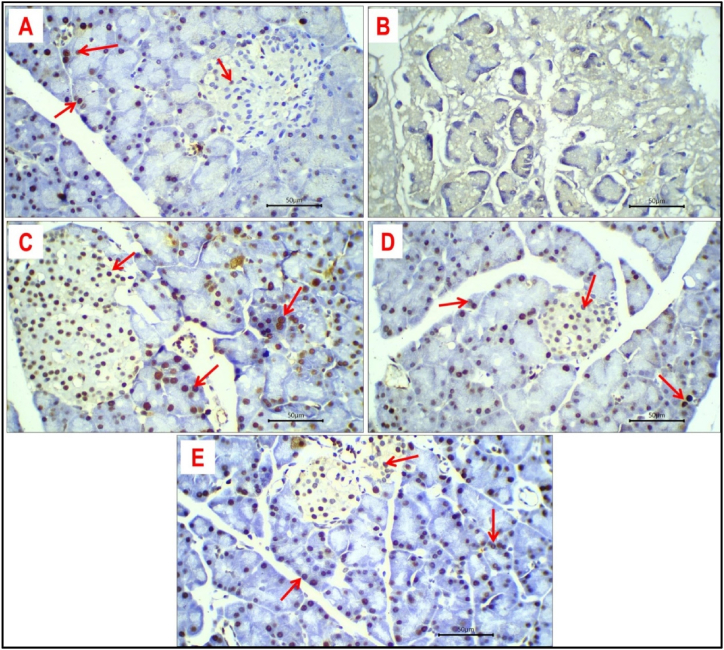
Expression of PCNA in pancreatic tissue of different study groups: Nuclear expression of PCNA (red arrows) in pancreatic tissue of control group **(A),** dexamethasone-treated group **(B)**, metformin-treated group **(C),** curcumin-treated group **(D)** and nano curcumin-treated group **(E).** Immune-stained sections; magnification is ×400 for all.

Expression of PCNA was detected as brown nuclear staining in positive cells. In general, expression of PCNA is moderate to strong (Fig. 10). Additionally, expression of PCNA was relatively higher in exocrine pancreatic acini compared to islets' cells. In different investigated groups, expression of PCNA was negative in the pancreatic tissue of dexamethasone-treated rats (Fig. 10 B) due to extensive necrosis of pancreatic tissue. The average percentage of PCNA-positive cells was 30 %, 75 %, 55 %, and 45 % in the pancreatic tissue of the control group (Fig. 10 A), metformin-treated group (Fig. 10C), curcumin-treated group (Fig. 10 D), and nano curcumin-treated group (Fig. 10 E); respectively.

Histopathological results demonstrated that both curcumin and curcumin NPs significantly reduced liver and pancreatic tissue damage, but curcumin NPs showed fewer residual inflammatory changes and necrosis. The reduced TNF expression (inflammation marker) and enhanced PCNA expression (cellular repair marker) further support curcumin's NPs regenerative potential. Calculating effect sizes for TNF and PCNA expression differences among groups could illustrate the degree of tissue recovery provided by curcumin NPs. Incorporating effect sizes and confidence intervals into the statistical analysis would not only strengthen the clinical significance of these findings but also clarify the magnitude of curcumin's NPs benefits relative to both untreated diabetic states and metformin.

## 4 Discussion

Insulin resistance is one of the potential pathways for the development of adult-onset diabetes, according to several theories. Insulin resistance induced by dexamethasone causes hyperinsulinemia, hyperglycemia, dyslipidemia, hepatic steatosis, muscle weakness, and body weight loss. Insulin resistance occurs prior to the manifestation of symptoms. Higher doses of dexamethasone are used in several therapeutic conditions. Insulin resistance can be treated early to stop the emergence of further issues [48].

In general, glucocorticoids (GCs) raise blood sugar levels through a variety of processes, including enhanced hepatic glucose synthesis (gluconeogenesis), decreased peripheral glucose absorption into muscle and adipose tissue, and breakdown of muscle and fat to supply extra substrates for glucose synthesis; Long-term exposure to GCs is linked to the development of severe insulin resistance and metabolic dysfunction; however, the exact biochemical mechanism underlying this association remains unclear [49]. The current investigation supports these results by showing that dexamethasone markedly raised insulin and blood glucose levels in insulin-resistant rats.

Due to its high metabolism, quick excretion from the body, and poor absorption, curcumin has a low bioavailability [50]. For simulating drug delivery to target organs, the nanosized particle size is helpful. In general, drugs that employ nanoparticle technology have poor oral solubility and bioavailability [51]. Nanosized particles can improve a drug's stability, bioavailability, and absorption [52].

In the current work, curcumin was loaded onto chitosan-TPP nanoparticles using the ionotropic gelation process. The TEM analysis of CUR NPs showed a median diameter of 68.75 nm and a spherical shape. The loading capacity and encapsulation efficiency were found to be 52.87 % and 96.67 %, respectively. According to FTIR studies, the hydroxide groups of curcumin and the ammonium groups of chitosan bind CUR and chitosan together more.

In the current investigation, the treatment of dexamethasone caused insulin resistance, which in turn caused diabetes, dyslipidemia, hepatic steatosis, muscle weakness, and body weight loss. The administration of curcumin and curcumin nanoparticles (100 mg/kg/orally) resulted in a considerable reduction in high serum glucose, insulin, and lipid levels and elevation in muscles and body weight. Additionally, the liver and pancreas pathological abnormalities caused by dexamethasone were ameliorated. Here, subcutaneous dexamethasone efficiently produced insulin resistance in normal rats as evidenced by elevated HOMA-IR index, hypertriglyceridemia, hyperglycemia, and hyperinsulinemia. Prior research demonstrated that rats could develop IR when exposed to dexamethasone at varying dose levels and for varying lengths of time. suppression of hepatic hexokinase activity, suppression of hepatic glucose oxidation, and promotion of hepatic gluconeogenesis are among the proposed mechanisms of dexamethasone-induced insulin resistance [53]. Insulin secretion and action are biologically related [54]. Insulin-related indicators, such as HOMA-IR, have been found in relation to insulin function in type 2 diabetes and the health of insulin-producing cells. The current study's findings suggest that by enhancing beta cell activity and lowering the insulin resistance index, CUR, and CUR NPs can improve type 2 diabetes. Mantzorou et al. showed that CUR reduced plasma glucose levels and enhanced insulin resistance in diabetic rats [55]. Additionally, CUR NPs supplementation significantly decreased FBS in comparison to the placebo in the Rahimi et al. research [56]. In a different investigation, CUR NPs (doses of 10 and 50 mg) decreased FBS by 32 % and 37 %, respectively, in rats with type 1 diabetes [57]. These outcomes are consistent with those of Ahmed et al. [58], who reported that treatment with Lut/ZnO NPs significantly decreased the levels of FBG, insulin, and HOMA-IR. These findings indicated that Lut/ZnO NPs successfully improved insulin sensitivity and glucose tolerance in rats with type 2 diabetes.

Another impact of dexamethasone that contributes to the development of insulin resistance in rats is the downregulation of GLUT4, Insulin-Mediated Induction A signaling mechanism may cause insulin-regulated GLUT4 translocation. That pathway involves lipid kinase phosphatidylinositol 3-kinase (PI3K). When insulin attaches to the insulin receptor on the surface of the target cell, the receptor changes shape, activating its tyrosine-kinase domain inside the cell. The proto-oncoprotein c-Cbl and insulin receptor substrates (IRS) are subsequently phosphorylated. The essential substrates in muscle and fat cells are IRS-1 and IRS-2. These substrates are found adjacent to the plasma membrane and attract effector molecules, including PI3K, which has been involved in the translocation of GLUT4 to the plasma membrane [59]. While Stimulation Mediated by Non-Insulin In skeletal muscle, GLUT4 translocation to the plasma membrane is stimulated by physical exercise. A mechanism other than PI3K, which is required for the insulin-stimulated pathway, is responsible for this activation. To fulfill the increased energy demands of skeletal muscle during exercise, skeletal muscle contraction triggers 5′-AMP-activated protein kinase (AMPK), which is thought to translocate exercise-responsive GLUT4-containing shuttles to the cell surface to mediate glucose transport [59,60]**.** According to earlier research, dexamethasone raised the levels of free fatty acids in rats, which may decrease the expression of the GLUT-4 transporter in cell membranes, reducing glucose absorption and reducing glucose metabolism in regions responsible for storing glucose [61,62].

In our study, CUR and CUR NPs reduce hyperglycemia and ameliorate insulin sensitivity by increasing GLUT4. According to Zhang et al., CUR has been demonstrated to improve cellular glucose uptake by the promotion of GLUT4 translocation from intracellular compartments to the plasma membrane, hence improving insulin sensitivity in the muscle tissue of insulin-resistant rats. The result of our study is consistent with the result of this study [35].

The rats' body weight was significantly lower after receiving dexamethasone (1 mg/kg I.P. daily) for 14 days than it was for the normal control group (p < 0.001). Dexamethasone treatment has been demonstrated to cause skeletal muscular atrophy, which may explain weight loss, in addition to the breakdown of muscle proteins [63,64], as well as the inhibition of muscle protein synthesis [65]. As shown in Table 1 and Fig. 4, the group treated with CUR and CUR NPs observed a much greater gain in body weight than the Dexa-treated group (p < 0.001), the weight gain in these rats is due to an increase in muscle mass. This observation aligns with several earlier research works that used different experimental strategies. In particular, mice given dexamethasone showed reduced weight gain compared to those fed a normal or high-fat diet [66,67].

The current investigation demonstrated that daily oral administration of dexamethasone resulted in a statistically significant increase in fasting blood glucose levels when compared to the normal control group. When metformin, curcumin, and curcumin NPS were given, the fasting glucose levels decreased. Days 10 and 14 of the dexamethasone therapy showed an increase in fasting glucose levels. Surprisingly, blood glucose levels in the dexamethasone groups increased from around 128 mg/dL to about 144 mg/dL in just three days, as shown in Table 2. This finding agreed with a prior study that examined the impact of curcumin on serum FBG levels in rats with diabetes [68,69]. For 14 days, a daily dose of 100 mg/kg of CUR and CUR NPs was administered. By the end of the trial, both curcumin groups' fasting blood glucose levels significantly decreased as compared to the insulin resistance group (Dexa group).

The administration of glucocorticoids causes lipid disturbances; it raises triglycerides, total cholesterol, and LDL cholesterol levels and lowers HDL levels, which may be a secondary cause of dyslipidemia. The mechanisms behind glucocorticoid-induced dyslipidemia may include impaired LDL catabolism, increased lipoprotein lipase activity, and subsequent elevation of LDL level due to increased plasma insulin [70]. The present study found that treatment with CUR and CUR NPs significantly improved the altered lipid profile. Increased HDL levels and significantly reduced high CH, TG, and LDL were observed with CUR and CUR NPs (100 gm/kg/oral). The HDL level is raised in the current study by CUR and CUR NPs, which is consistent with previous research [71]. These elevated HDL levels will aid in reducing a variety of dyslipidemia-related cardiovascular problems. According to other studies that support our findings, CH, TG, and LDL were all markedly decreased by CUR and CUR NPs [[72], [73], [74]]. In the Rahimi et al. patients with T2DM received CUR NPs supplementation (80 mg/day) vs a placebo for three months. The findings demonstrated that CUR NPs supplementation substantially reduced triglycerides, cholesterol, and LDL when compared to placebo [75]. In a different study, glucose 6-phosphatase and phosphoenolpyruvate carboxykinase in the liver, as well as the regulation of the SREBP (sterol regulatory element-binding proteins) cycle, were found to be the causes of the effects of CUR on diabetic rats treated with doses of 40 and 80 mg/kg of the drug. The results indicated a decrease in serum levels of FBS, insulin, cholesterol, triglyceride, LDL, and insulin resistance in the rats treated with CUR [76]. The outcomes of these investigations align with the findings of our investigation. According to RM El-Gharbawy et al. [77], in Type-2 diabetes, zinc oxide nanoparticles restore abnormalities in lipid metabolism that generally lead to elevated serum lipid levels. These outcomes concur with the current study's conclusions.

Through a combination of increased fatty acid synthesis and impaired fatty acid β oxidation in the liver, glucocorticoids can contribute to the formation of fatty liver [78]. Elevated amounts of free fatty acids have been linked to the emergence of skeletal muscle insulin resistance, hypertension, and fatty liver, which is thought to represent the hepatic outcome of the metabolic syndrome because of hepatic insulin resistance [79]. The current investigation confirms the previous finding that the injection of dexamethasone (1 mg/kg/i.p.) caused the development of insulin resistance-related hepatic steatosis. The pathological alterations in the liver were significantly ameliorated by treatment with CUR and CUR NPs (100 mg/kg/orally) (Fig. 7; E and F). This could be the result of a reduction in circulating fatty acid levels, which would reduce the amount of fat dexamethasone-induced liver fat development.

The present study demonstrates that Dexa-induced hepatotoxicity is characterized by a significant increase in the serum activity of ALT, AST, and Albumin. These results are consistent with a recent study we conducted when rats given Dexa showed higher serum levels of liver marker enzymes [80]. These enzymes' activity are sensitive markers of liver damage and are correlated with the severity of the damage [81]. There were notable variations in the levels of albumin, ALT, and AST between the CUR and CUR NPS treatment groups, according to the statistical results. Because curcumin absorbs slowly when taken orally, CUR NPs at a dose of 100 mg/kg BW are more effective than curcumin at the same dose in lowering AST, and ALT levels and increasing albumin levels. As a result, curcumin that has been altered into nanoparticle form may be more effective at lowering and preventing the production of free radicals, resulting in decreased levels of AST, and ALT, and increased levels of albumin. Significant changes in histopathology further supported the damage of liver tissue caused by Dexa. degenerative alterations, such as inflammation, necrosis, cloudy swelling, granular and glassy cytoplasm, micro-vesicular and macro-vesicular steatosis, and focal congestion. Our findings are consistent with those of Safaei et al. [70]**,** who demonstrated that Dexa induced extreme hepatocyte degeneration, necrosis, and inflammatory cell infiltration. Gutiérrez et al. [82] revealed that selenium nanoparticle intake induced a beneficial effect on diabetic rats concerning liver function, as measured by a reduction in ALT, AST, and ALP. These data are in agreement with the results of the current study. Consuming CUR-loaded PLA–PEG NPs improved liver enzymes in diabetic rats, as indicated by a decrease in ALT, and AST according to El-Naggar et al. [83], These findings are consistent with the study's findings.

These findings suggested that prepared nanoparticles could be utilized to prevent or ameliorate diabetes. It has long been believed that oxidative stress is one of the primary damaging factors that cause the development of insulin resistance. Although the exact mechanism of oxidative stress production is still up for discussion, it has been suggested that several mechanisms are involved [84]. One of the primary causes of oxidative stress is reactive oxygen species (ROS), which are an unavoidable consequence of metabolism. The primary process that produces ROS is the passing of electrons from the respiratory chain of the mitochondria and their subsequent transfer to molecular oxygen, which forms the superoxide anion (O2−). ROS is the O2 produced when the enzyme NADPH oxidase is activated [85]. Increased auto-oxidative and non-enzymatic glycosylation are among the potential mechanisms that significantly trigger the formation of free radicals and radical-induced lipid peroxidation [86] Pro-oxidative conditions are caused by increased ROS production, which throws off the equilibrium between oxidant and antioxidant status levels.

The average tissue MDA level in the pancreas and liver increased in the current study when the Dexamethasone group was included. Elevations in MDA levels are indicative of increased lipid peroxidation, which is evidence that glucocorticoid medication causes oxidative stress. Compared to the group that received CUR, the treatment group that received CUR NPs exhibited the lowest levels of MDA. This is because curcumin that has been transformed into nanoparticles has a higher bioavailability, which enables it to be better absorbed by the body, reach its intended organs, and lower liver and pancreas tissue MDA levels. By elevating GPx activity and lowering elevated liver MDA levels, nanoparticles help to promote curcumin's adsorption in intestinal epithelial cells and enhance its hepatoprotective effects in rats [87]. A prior study that estimated the antioxidant and antihyperglycemic effects of Allium boonei extract in rats with dexamethasone-induced hyperglycemia revealed similar results. Daily injection of dexamethasone (0.4 mg/kg) for 30 days to produce Hyperglycemia resulted in a notable increase in the MDA level [88].

The liver's accumulation of lipids may oxidize, releasing free radicals such as reactive oxygen species (ROS) [89]. By destroying unsaturated fatty acids in cell membranes, ROS causes lipid peroxidation and decreases endogenous antioxidants, which damages the liver [90]. Glutathione (GSH), which is a substrate for glutathione peroxidase (GPx) and glutathione S-transferases (GST), is the first line of defense against free radicals. It is responsible for replenishing GPXs, which detoxify lipid hydroperoxides and H_2_O_2_ [91]. In this study, the liver and pancreatic GSH levels were decreased in the Dexa-treated group compared to the normal control group. In contrast, CUR NPs therapy led to a greater elevation in pancreatic and liver GSH levels than did CUR. In accordance with these results, Lv et al., 2018 reported that Dexa treatment reduced glutathione peroxidase levels in broiler liver [92]**.**

Hepatic and pancreatic tissues of SOD activity increased in animals treated with metformin, CUR, and higher in CUR NPs, compared to the diabetic control group. This increase was more important in the liver and pancreas. Karihtala and Soini [93] suggests that SOD readily hydrolyzes hydrogen peroxide by disproportionation damaging superoxides. By increasing the synthesis of SOD, an enzyme essential to the body's antioxidant protection, CUR, and CUR NPs function greater than the reference drug (metformin), especially CUR NPs.

Our results with diabetic rats demonstrated that our available diabetes treatment options, particularly 100 mg/kg CUR NPs, effectively restored the activities of antioxidant enzymes in the pancreas and liver along with reducing oxidative stress markers, MDA, and increasing GSH and SOD in the hepatic and pancreatic tissues. Additionally, curcumin can increase overall antioxidant activity, improve islet viability, and reduce the generation of reactive oxygen species (ROS) in islets, hence restoring pancreatic islets [94,95]. Curcumin's ability to scavenge free radicals by interacting with the oxidative cascade to reduce oxidative enzymes, restore the antioxidant status, and chelate metal ions was linked to its ameliorative effect on hepatic GSH levels, which prevented the Fenton reaction [96].

Histological investigations of the liver and pancreas conclude that dexamethasone induces significant liver and pancreas damage in rats, characterized by necrosis, inflammation, and steatosis. Treatment with metformin mitigates these effects to a moderate extent, while curcumin and nano-curcumin offer substantial protection, preserving hepatic architecture and significantly reducing pathological changes. These findings highlight the potential of curcumin and nano-curcumin as effective therapeutic agents against dexamethasone-induced liver and pancreas damage.

Limitations of the current study include: **1)** The use of animal models to study type 2 diabetes (T2DM) has limitations, as the physiology and metabolic responses in animals may not fully replicate those in humans. This can affect the generalizability of the results to human disease mechanisms and treatment responses. **2)** A small sample size reduces the statistical power of the study, making it difficult to detect significant effects or accurately estimate variation within groups. It also limits the ability to generalize the results to a broader population. **3)** Short study duration does not fully capture the long-term effects of interventions or assess the progression of T2DM. Chronic conditions such as T2DM require longer observation periods to understand the sustained effect of treatments. **4)** Lack of sample diversity, as a homogeneous sample (e.g., age, gender, genetic background) limits the applicability of the results to diverse populations and may not reflect varying responses due to genetic, lifestyle, or environmental factors. **5)** Experiments are conducted in vitro, the results may not fully mimic the conditions of the in vivo, where complex interactions and regulatory systems influence cellular responses. These limitations highlight the need for further research using larger, more diverse samples, human models, and extended study periods to confirm and extend these findings.

## 5 Conclusion

This study makes a significant novel contribution to the field of diabetes research by highlighting the potential of nanoparticle-based formulations, particularly nano-curcumin, as an advanced treatment strategy for T2DM. By enhancing the bioavailability and effectiveness of curcumin, the nano-formulation addresses the limitations of conventional curcumin supplementation, which is often limited by poor absorption. Based on the study's findings, nano-curcumin offers significant potential for clinical application in human diabetes treatment. The nano-formulated curcumin demonstrated enhanced efficacy in lowering fasting blood glucose, improving lipid profiles, and reducing liver and pancreas tissue damage. Its antioxidant properties, particularly the increased SOD and GSH and reduced MDA levels, suggest a protective effect against oxidative stress. These results support nano-curcumin's viability as an adjunct therapy for T2DM, potentially complementing traditional treatments by improving metabolic control and reducing diabetic complications.

## CRediT authorship contribution statement

**Amany M. Hamed:** Writing – review & editing, Writing – original draft, Validation, Software, Resources, Methodology, Formal analysis, Data curation, Conceptualization. **Dalia A. Elbahy:** Writing – review & editing, Software, Resources, Data curation. **Ahmed RH. Ahmed:** Writing – review & editing, Writing – original draft, Validation, Resources, Methodology, Data curation. **Shymaa A. Thabet:** Writing – original draft, Methodology, Data curation. **Rasha Abdeen Refaei:** Writing – original draft, Validation, Software, Resources, Methodology, Data curation. **Islam Ragab:** Writing – review & editing, Validation, Software. **Safaa Mohammed Elmahdy:** Writing – review & editing, Visualization, Validation, Resources, Methodology, Investigation, Formal analysis, Data curation. **Ahmed S. Osman:** Writing – review & editing, Visualization, Software, Formal analysis, Data curation. **Azza MA. Abouelella:** Writing – review & editing, Writing – original draft, Methodology, Data curation, Conceptualization.

## Consent to participate

Not applicable.

## Data availability

All data generated or analyzed during this study are included in this published article.

## Consent for publication

The manuscript is original. It has not been published previously by any of the authors and even not under consideration in any other journal at the time of submission.

## Funding

The research has not received external funding

## Declaration of competing interest

The authors declare that they have no known competing financial interests or personal relationships that could have appeared to influence the work reported in this paper.

## Abbreviations

**T2DM**: type 2 diabetes mellitus
**CUR**: curcumin
CUR NPs: curcumin nanoparticles'
**NPs**: nanoparticles
**Dexa**: dexamethasone
**DPX**: Dibutyl phthalate Polystyrene Xylene
**DMSO**: Dimethyl sulfoxide
**GCs**: glucocorticoids
**NAFLD**: non-alcoholic fatty liver disease
**NASHD**: non-alcoholic steatohepatitis disease
**NASH**: non-alcoholic steatohepatitis
**HOMA-IR**: insulin resistance
**HDL**: high-density lipoproteins
**LDL**: low-density lipoproteins
**GSH**: glutathione
**SOD**: superoxide dismutase
**MDA**: malondialdehyde
**ROS**: reactive oxygen species
**GPx**: glutathione peroxidase
**GLUT-4**: glucose transporter-4
**TPP**: tripolyphosphate
**TNF**: Tumor necrosis factor
**PCNA**: Proliferating Cell Nuclear Antigen
**PLA-PEG**: polylactide-poly (ethylene glycol)
**Lut/ZnO NPs**: Luteolin loaded on zinc oxide nanoparticles
**GLP-1**: Glucagon-like peptide-1

## Supplementary data

This article doesn't contain supplementary data.
